# Editorial: Phytochemicals in modern medicine: health benefits and toxicological considerations

**DOI:** 10.3389/fmed.2026.1835975

**Published:** 2026-04-17

**Authors:** Cristian Scheau, Aleksandra Buha Djordjevic, Brankica Filipic, Constantin Caruntu

**Affiliations:** 1 Department of Physiology, the “Carol Davila” University of Medicine and Pharmacy, Bucharest, Romania; 2 Department of Radiology and Medical Imaging, “Foisor” Clinical Hospital of Orthopaedics, Traumatology and Osteoarticular TB, Bucharest, Romania; 3 Department of Toxicology, Faculty of Pharmacy, University of Belgrade, Belgrade, Serbia; 4 Department of Microbiology and Immunology, Faculty of Pharmacy, University of Belgrade, Belgrade, Serbia; 5 Department of Dermatology, “Prof. N.C. Paulescu” National Institute of Diabetes, Nutrition and Metabolic Diseases, Bucharest, Romania

**Keywords:** clinical translation, drug delivery systems, immune regulation, natural bioactive compounds, oxidative stress, phytochemical extraction, phytochemicals

Natural compounds are increasingly studied as sources of biologically active agents for various conditions, including inflammatory, infectious, and neoplastic diseases, and significant pharmaceutical innovations and therapeutic applications have promoted their clinical translation (1). Advances in phytochemistry and molecular biology have improved the understanding of the action mechanisms of a variety of compounds (2). Within the complexity of their effects, phytochemicals showcase strong and diverse biologic activity, which includes anti-inflammatory, anti-infectious, antioxidant, and antiproliferative properties (3). Nevertheless, the path toward clinical application is hindered by the variability in plant sources, extraction procedures, formulation protocol, and study design. The articles collected in this Research Topic provide a multidisciplinary perspective on how these bioactive compounds can be extracted, evaluated, and considered for translation into clinical practice.

One of the critical steps in the development and implementation of novel compounds derived from natural products is the safe and effective extraction and preservation of the bioactive molecules. In this regard, Sun et al. have provided a comprehensive overview on the extraction methods and their potential influence on the composition and activity of plant-derived mixtures. The authors emphasize the critical parameters influencing the stability of bioactive compounds, which are the solvent polarity, particle size, extraction time and temperature. Furthermore, novel technologies shorten processing time, improve extraction efficiency, and increase preservation of bioactive molecules. Moreover, this area still faces significant methodological challenges, as reproducibility remains limited. This is caused by the differences in phytochemical formulations due to variability of plant sources, harvesting protocols, methods and parameters of extraction, storage and post-processing conditions. Therefore, biological activity may differ consistently between formulations leading to difficulties in comparisons across studies and inconsistent outcomes. As such, progress in the extraction technology should be doubled by detailed and thorough characterisations as well as standardized reporting, in order to allow for rigourous assessments and safe transition toward patients.

Further contributions to the Research Topic explore the pharmacological roles and clinical applications of natural compounds in oncology. Phytochemicals continue to attract interest in cancer research owning to their ability to modulate inflammation, oxidative stress and tumor proliferation. through multiple converging mechanisms (4, 5). However, solid preclinical data and rationale are not always translated into reliable clinical benefits. This is particularly evident for curcumin, a polyphenolic compound with known biological activity including antioxidant and antiproliferative effects, previously demonstrated to be useful in a variety of malignancies (6). Despite its advantages, its clinical applicability is limited by poor bioavailability, water solubility, and rapid metabolism (7, 8). In light of these conditions, Sanlier et al. provided a detailed review of the potential applications of curcumin in gynaecologic diseases, which is particularly relevant because the authors highlight further progress in compound formulation strategies such as nanoparticles, liposomes, and lipid-based delivery systems which can provide significant support for curcumin but also for other similar cases (8). The development of new agents has prompted adoption of drug-delivery approaches from various related fields, with 3D-printing, hydrogel systems, and cyclodextrin complexes representing innovations aimed at improving delivery (9–11).

Another relevant dimension explored in the Research Topic is the role of natural products in infectious diseases, particularly in the context of COVID-19. Dauth et al. have reported the findings of a prospective randomized pilot study which investigated the effects of a thyme-ivy syrup in COVID-19 patients and observed variations of several cytokines that may suggest potential immunomodulatory effects. COVID-19 determines numerous complications with system-wide impact; these can be attributed to direct viral effects and the associated inflammatory environment (12, 13). In their systematic review and meta-analysis, Prabhakornritta et al. assesses the clinical evidence on the use of *Andrographis paniculata*-derived compounds in COVID-19. The authors identified important trends suggesting the potential anti-inflammatory and immunomodulatory effects and emphasized the need for rigor and standardization in the design of clinical trials in order to definitively establish the efficacy of these compounds in the treatment of viral infections. Taken together, these studies confirm the therapeutic potential of natural compounds but underline the current limitations pertaining to heterogeneous formulations and small datasets, with uncertainty in replicating the results in other experimental settings. The complexity of post-viral sequelae and the variety of reported abnormalities throughout the course of the disease underline the necessity of targeting these diverse mechanisms, while also considering the safety and potential toxicological implications (14). Natural compounds should not be considered intrinsically safe, as dose-dependent adverse effects, variable purity, interactions with concomitant drugs or contamination risks are realistic concerns (15). Data related to long-term safery and regulatory assessment of these products remains scarce, and these issues should be integrated more explicitly into future studies.

Collectively, the contributions to this Research Topic highlight several important themes of what are otherwise very diverse applications of phytochemicals. The modulation of oxidative stress and inflammatory/immune pathways are key features, alongside the necessity for continual improvement of the stability and quality of the phytochemical preparations. Another common theme of the abovementioned papers is the heterogeneity in formulations and extraction procedures, as well as the pharmacokinetic contraints and limited standardization; the still existing gap between promising experimental findings and fragmented clinical evidence remains a topic of concern.

Future research should therefore go beyond simple demonstration of isolated biological effects and prioritize the harmonization of extraction methods and characterization protocols. Also, improved regulatory clarity and integration of toxicological assessments are necessary for conducting large-scale clinical trials on the safety and efficacy of phytochemicals in diverse patient populations, as a step to incorporating these substances into evidence-based clinical medicine.

Overall, the studies showcased in this Research Topic contribute valuable knowledge to this rapidly evolving field and reiterate the growing role of natural compounds in modern biomedical research within a more rigorous translational framework.
